# Spatial Distribution and Ecological Determinants of Coexisting Hybrid Oak Species: A Study in Yushan’s Mixed Forest

**DOI:** 10.3390/plants13071000

**Published:** 2024-03-30

**Authors:** Xuan Li, Yongfu Li, Yousry A. El-Kassaby, Yanming Fang

**Affiliations:** 1 Co-Innovation Center for Sustainable Forestry in Southern China, Key Laboratory of State Forestry and Grassland Administration on Subtropical Forest Biodiversity Conservation, College of Life Science, Nanjing Forestry University, 159 Longpan Road, Nanjing 210037, China; xuanli18851128817@163.com; 2 Department of Forest and Conservation Sciences, Faculty of Forestry, The University of British Columbia, 2424 Main Mall, Vancouver, BC V6T 1Z4, Canada; 3 Jiangsu Key Laboratory for the Research and Utilization of Plant Resources, Institute of Botany, Jiangsu Province and Chinese Academy of Sciences, Nanjing 210014, China; yongfuli@njfu.edu.cn

**Keywords:** *Quercus*, oak, spatial coexistence, ecological traits, nutritional elements

## Abstract

Ecological niche partitioning is crucial in reducing interspecific competition, fostering species coexistence, and preserving biodiversity. Our research, conducted in a hybrid mixed oak forest in Yushan, Jiangsu, China, focuses on *Quercus acutissima*, *Q. variabilis*, *Q. fabri*, and *Q. serrata* var. *brevipetiolata*. Using Point Pattern Analysis, we investigated the spatial relationships and ecological trait autocorrelation, including total carbon (TC), nitrogen (TN), phosphorus (TP), potassium (TK), and breast height diameter (DBH). Our findings show aggregated distribution patterns within the oak populations. The Inhomogeneous Poisson Point model highlights the impact of environmental heterogeneity on *Q. variabilis*, leading to distinct distribution patterns, while other species showed wider dispersion. This study reveals aggregated interspecific interactions, with a notable dispersal pattern between *Q. acutissima* and *Q. variabilis*. We observed significant variability in nutrient elements, indicating distinct nutrient dynamics and uptake processes. The variations in total carbon (TC), nitrogen (TN), phosphorus (TP), and potassium (TK) suggest distinct nutrient dynamics, with TK showing the highest variability. Despite variations in TC, TK, and TP, the species did not form distinct classes, suggesting overlapping nutritional strategies and environmental adaptations. Furthermore, spatial autocorrelation analysis indicates strong positive correlations for DBH, TC, and TP, whereas TK and TN correlations are non-significant. The results suggest habitat filtering as a key driver in intraspecific relationships, with a finer spatial scale of ecological niche division through TC and TP, which is crucial for maintaining coexistence among these oak species.

## 1. Introduction

The spatial distribution and dynamics of plant communities are intricately related to ecological mechanisms encompassing habitat heterogeneity, seed dispersal limitation, organism interactions, and anthropogenic disturbances [1,2,3,4,5]. These mechanisms not only facilitate long-term species coexistence but also intensify interspecific competition and foster favorable growth environments [6]. Along with ecological mechanisms, introgressive hybridization plays an important role in tree coexistence [7,8]. A range of evolutionary and ecological consequences of hybridization and introgression among oak trees have been reported, including community reorganization and restructuring [8,9,10]. Introgression can result, over time, in the fusion of distantly related species, preventing coexistence and leading populations to disperse excessively [11]. Therefore, this study of hybrid mixed forests contributes to a deeper understanding of forest-forming species’ spatial distribution and coexistence.

Ecological elements are essential for plant growth and development and play key roles in ecosystem nutrient cycling and energy flow [12,13]. Existing research has confirmed that variations in ecological nutrient elements like carbon, nitrogen, phosphorus, and potassium are influenced by a combination of environmental factors and genetic makeup [14]. For instance, carbon is primarily fixed by plants during photosynthesis, whereas nitrogen, phosphorus, and potassium are crucial for protein synthesis, energy transformation, and cellular physiological processes, respectively [15,16,17]. The availability and cycling of these elements directly impact plant growth strategies and adaptability to environmental conditions [18,19,20] and their strategies in resource acquisition, utilization, and response to environmental stresses, thereby illuminating how ecosystem diversity and stability are maintained.

Ecologists have developed methods to study the distribution patterns of plant populations at different scales, such as trend surface analysis for large-scale patterns, two-term local variance for medium-scale patterns, and species juxtaposition for small-scale patterns [21,22,23]. Point Pattern Analysis (PPA) enables not only large-scale forest dynamics monitoring [24] but also small-scale population dynamics, revealing the driving mechanisms of local plant community spatial patterns [25]. The selection of appropriate statistical function(s) and the choice of appropriate null models for different scientific problems are important in PPA [26]. For ecologists, the aforementioned analytical methods are active study issues. Furthermore, we know that the distribution of species exhibits some correlation, and spatial autocorrelation analysis is very important [27]. However, a research gap exists in the spatial autocorrelation of ecological traits in forest ecosystems, particularly in mixed forest populations composed of multiple species at small scales such as oak populations.

*Quercus* species are vital components of China’s deciduous broad-leaved forests [28]. In our extensive research involving long-term sampling and examination of their geographical distribution, we observed that four oak species—*Quercus acutissima*, *Q. variabilis*, *Q. fabri,* and *Q. serrata* var. *brevipetiolata*—are prominently established within these woodlands, exhibiting partial sympatric distribution points. Analysis of genetic diversity and variation in *Quercus* populations, both at large and local scales, showed that there are many introgressions among these four oak species [29,30]. Thus, from an evolutionary perspective, studying the spatial distribution characteristics of hybrid mixed forests composed of multiple oak species not only helps in revealing the interaction between species and environment but also helps in understanding species distribution patterns and coexistence.

In this study, we focus on a mixed forest in Yushan, comprising four oak species—*Q. acutissima* Carruth, *Q. variabilis* Blume, *Q. fabri* Hance, and *Q. serrata* var. *brevipetiolata* (A. DC.) Nakai. Our preliminary molecular work has confirmed introgressive hybridization among these species [29,30]. In this study, we delve into the intra- and interspecific spatial relationships of these oaks using Point Pattern Analysis (PPA). Subsequently, we analyze the spatial distribution of key nutritional elements (TC, TN, TP, and TK) within these trees. By conducting spatial autocorrelation analysis, we link these distribution patterns to the nutritional elements, aiming to understand how these species coexist, compete, and cooperate for resources and respond to environmental stresses. This comprehensive approach offers new insights into the coexistence and distribution patterns among the oak species in Yushan’s mixed forest, contributing significantly to the scientific knowledge needed for effective forest management and conservation.

## 2. Results

### 2.1. Intraspecific and Interspecific Distribution Patterns

**Figure 1:**
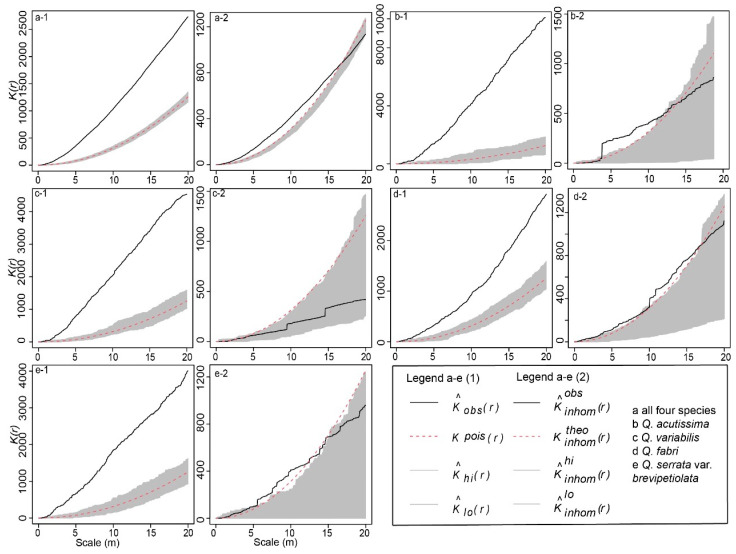
Spatial point pattern of four oaks in the mixed forests. (**a(1–2)**) all oak species; (**b(1–2)**) *Q. acutissima*; (**c(1–2)**); *Q*. *variabilis*; (**d(1–2)**) *Q*. *fabri*; (**e(1–2)**) *Q*. *serrata* var. *brevipetiolata* (Note: The gray part is the 99% confidence interval composed of the upper and lower envelope traces, the red dotted line represents the theoretical value, and the black realization represents the measured value).

**Figure 2:**
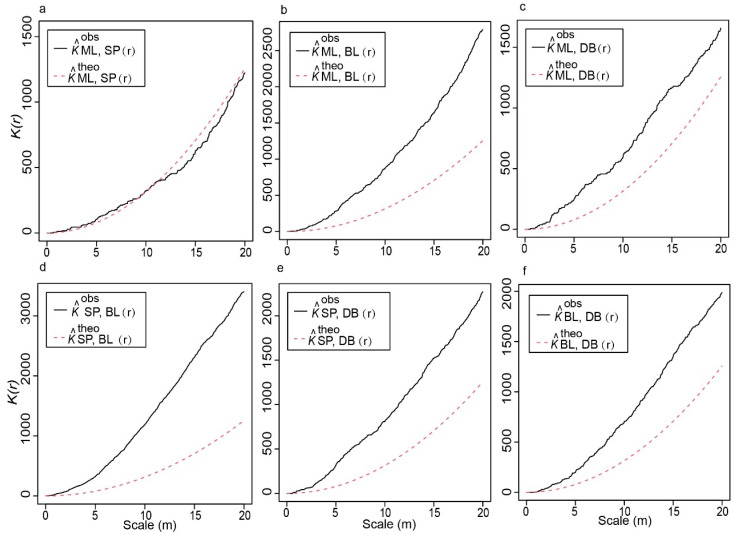
Spatial point pattern of four oaks in the mixed forests. (**a**) *Q. acutissima*–*Q*. *variabilis*; (**b**) *Q. acutissima*–*Q*. *fabri*; (**c**) *Q. acutissima*–*Q*. *serrata* var. *brevipetiolata*; (**d**) *Q*. *variabilis*–*Q*. *fabri*; (**e**) *Q*. *variabilis*–*Q*. *serrata* var. *brevipetiolata*; (**f**) *Q*. *fabri*–*Q*. *serrata* var. *brevipetiolata*.

Figure 1 illustrates the spatial point patterns of four oak species in the mixed forests of Yushan, as revealed by our Point Pattern Analysis (PPA). Figure 1a(1–2) shows the overall distribution of all oak species combined. Using the Complete Spatial Randomness (CSR) model, we observed a strong aggregation in their distribution, indicated by *K*(r) values exceeding theoretical values and the wrap-around line (Figure 1(a-1)). Conversely, under the Inhomogeneous Poisson Process (IPP) model, these populations gradually transitioned to a random distribution beyond 16 m (Figure 1(a-2)). In the individual species analysis, *Q. acutissima* and *Q. variabilis* exhibited an increasing clustering trend with distance under the CSR model (Figure 1(b-1)), whereas *Q. variabilis* showed a discrete distribution pattern under the IPP model (Figure 1(c-2)). *Q. fabri* and *Q. serrata* var. *brevipetiolata* displayed similar patterns with their populations diverging after 15–16 m (Figure 1(d-2),(e-2)).

We display the outcomes of a bivariate Point Pattern Analysis (PPA), which explores the spatial relationships between pairs of oak species in Yushan’s mixed forests (Figure 2). This analysis provides insights into how these species pairs are spatially associated with each other. We discovered that at around 11 m, the interspecific relationships between *Q. acutissima* and *Q. variabilis* were identical; however, after 11 m, they became scattered, and as the distance increased, they became similar again. (Figure 2a). The remaining five pairs of interspecific relationships are all characterized by aggregate distribution. With increasing distance, the trend of aggregation between *Q. acutissima* and *Q. serrata* var. *brevipetiolata* declined. (Figure 2c). The interspecific relationship between *Q. fabri* and *Q. variabilis*, on either hand, is the total opposite of the former, with an aggregated distribution of the increasing distance between the two species (Figure 2d). At close distances of 0–10 m, the intensity of aggregation distribution between *Q. variabilis* and *Q. serrata* var. *brevipetiolata* was higher than that of the remaining four species pairs (*Q. acutissima*–*Q. fabri*, *Q. acutissima*–*Q. serrata* var. *brevipetiolata*, *Q. variabilis*–*Q. fabri*, and *Q. fabri–Q. serrata* var. *brevipetiolata*).

### 2.2. Size Class Analysis

**Figure 3:**
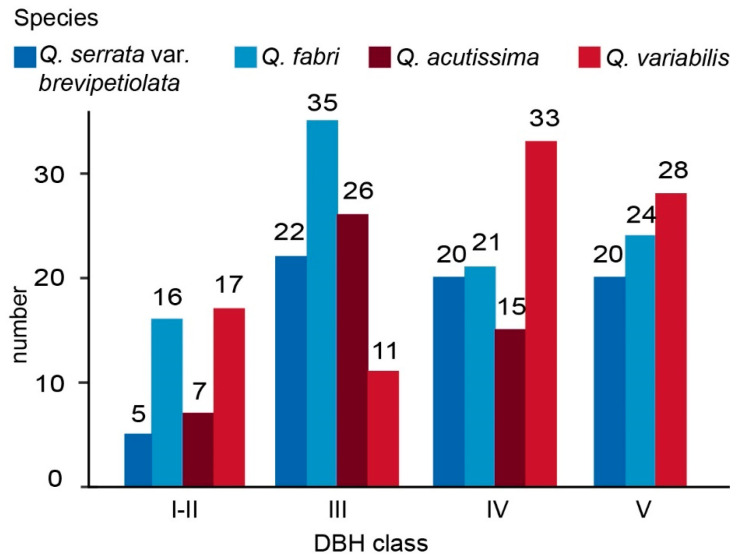
DBH class structure of four oak tree populations. DBH class: 0–2.5 cm (I–II); 2.5–7.4 cm (III); 7.5–22.5 cm (IV); DBH > 22.5 cm (V).

In our study, we analyzed the Diameter at Breast Height (DBH) of four oak species in Yushan’s mixed forest. Table 1 presents a summary of the DBH data, showing that the average DBH for the combined oak population is 13 cm, with a maximum of 38.4 cm. We ranked the four oak populations in the following order: *Q. variabilis* > *Q. serrata* var. *brevipetiolata* > *Q. fabri* > *Q. acutissima*. The distribution of DBH classes among the species, as illustrated in Figure 3, reveals distinct patterns. According to Zhongxiang Qu’s classification of standing trees and seedlings (Class I (seedlings): H (height) < 0.33 m; Class II (young trees): H > 0.33 m, DBH < 2.5 cm; Class III (standing trees): 2.5 < DBH ≤ 7.4 cm; Class IV (standing trees): 7.4 < DBH ≤ 22.5 cm; Class V (standing trees): DBH > 22.5 cm). *Q. acutissima* has a DBH primarily in the III and IV classes (2.5–22.5 cm), with no individuals exceeding class V (DBH > 22.5 cm); *Q. variabilis* predominantly falls within the IV and V classes, *Q. fabri*’s DBH distribution spans from classes II to V (0.5–38.4 cm), with a substantial proportion in the III class, accounting for 36.5% of its population; *Q. serrata* var. *brevipetiolata* shows an even distribution across classes III, IV, and V.

### 2.3. Variation Analysis of Leaf Functional Traits

**Figure 4:**
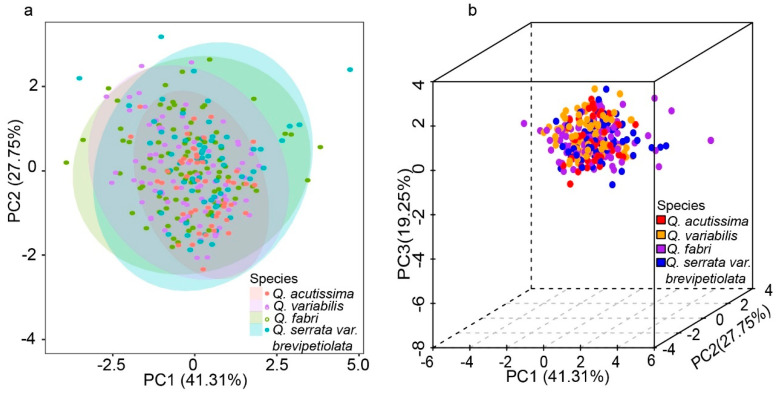
Principal component analysis of leaf functional traits of four *Quercus* species. (**a**) Two-dimensional principal component analysis; (**b**) Three-dimensional principal component analysis.

In our analysis of leaf functional traits, as detailed in Table 2, the mean values of total carbon (TC), nitrogen (TN), phosphorus (TP), and potassium (TK) for the overall oak population were found to be 296.85 ± 72.59, 18.12 ± 6.78, 0.83 ± 0.34, and 5.10 ± 2.42 mg g^−1^, respectively (Table 2). Notably, the order of magnitude in variation among these nutrient elements was observed to be highest for TK (15.7-fold), followed by TP (15.0-fold), TN (11.6-fold), and TC (5.0-fold), indicating distinct nutrient dynamics within the oak species. The Least Significant Difference (LSD) multiple comparison method revealed significant differences in TC, TK, and TP levels among the four oak species, except TN. TK, in particular, showed marked variability across the species, suggesting varied nutrient uptake or metabolic processes among them. Furthermore, the two- and three-dimensional Principal Component Analysis (PCA), as illustrated in Figure 4, revealed that the four oak species did not segregate into distinct classes based on these nutrient elements. This finding implies a complex interplay of nutritional factors within the oak species in the mixed forest, suggesting overlapping nutritional strategies or environmental adaptations (Figure 4). Moreover, nutritional strategies may be influenced by a variety of factors, including the size of the trees and their spatial distribution, not just species differences.

### 2.4. Spatial Autocorrelation Analysis of DBH and Leaf Functional Traits

**Figure 5:**
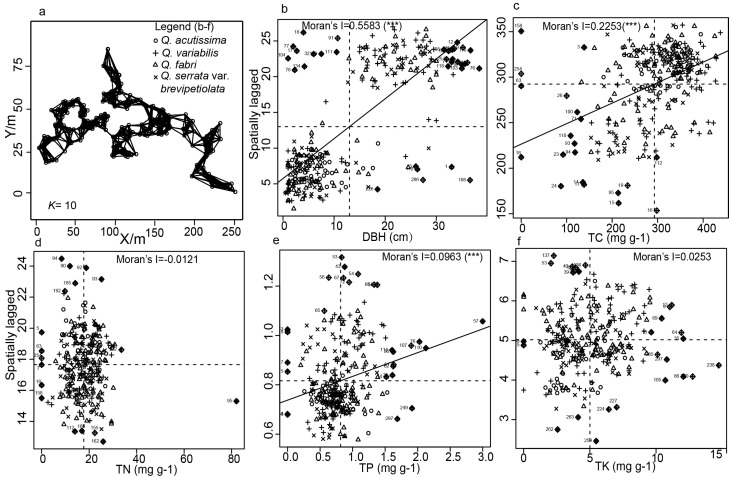
Local Moran test of five ecological indicators. (**a**) Distance-based neighbors; (**b**) Moran scatter chart of DBH; (**c**) Moran scatter chart of TC; (**d**) Moran scatter chart of TN; (**e**) Moran scatter chart of TP; (**f**) Moran scatter chart of TK. Note: The solid diamond indicates that this point has an overlap of two species, encased within a diamond border. ***: *p* < 0.001.

In our spatial autocorrelation analysis, represented in Table 3 and visualized in Figure 5, we focused on Moran’s I for various ecological indicators. The Moran’s I values for DBH, TC, TP, and TK all were between 0 and 1. Specifically, DBH, TP, and TC demonstrated significant positive spatial correlations, indicating a tendency for similar values to cluster spatially. In contrast, TK did not exhibit a significant correlation, suggesting a more random spatial distribution among the oak species (Table 3). Additionally, Moran’s I for TN was −0.0121, illustrating a non-significant negative spatial correlation (*p* = 0.6536), indicating no clear pattern in the spatial distribution of nitrogen. To further dissect these patterns, we conducted local tests, as shown in Figure 5, to identify clusters of similar values and specific points of distribution. The scatter plots in the figure, divided into four quadrants (low-low, low-high, high-low, and high-high), illustrate the local spatial correlations. For DBH, the majority of *Q. variabilis* and *Q. serrata* var. *brevipetiolata* individuals clustered in areas with high DBH values, indicating that larger trees are more likely to be found near each other. Conversely, *Q. acutissima* and *Q. fabri* predominantly clustered in areas with smaller DBH values (Figure 5b). In the case of TC, a notable aggregation was observed at higher concentrations, predominantly among *Q. fabri* and *Q. variabilis* individuals (Figure 5c), with *Q. fabri* and *Q. variabilis* individuals predominating. The spatial pattern for TP contrasted with that of TC, with a different set of species, including *Q. variabilis*, *Q. acutissima*, and *Q. fabri*, showing predominance. For TK, the local spatial correlation lacked significant clustering. The negative spatial correlation for TN was also non-significant, and no distinct distribution pattern was evident for the four oak species in the local.

## 3. Discussion

### 3.1. Spatial Distribution Patterns of Oak Mixed Forest

Aggregated, random, and discrete are the three states of species’ spatial distribution. Based on the CSR analysis, the *K*(r) statistical function revealed that all four oak populations exhibited an aggregated distribution. *Q. acutissima* and *Q. serrata* var. *brevipetiolata* showed greater aggregation as distance increased, whereas *Q. fabri* and *Q. variabilis* showed decreased aggregation. The decreasing trend of *Q. variabilis* is not so obvious. Ecological mechanisms, such as environmental heterogeneity, mostly caused these patterns in natural environments, acorn dispersal ability, density constraints, and inter-organismal interactions [1,2,23]. Abiotic factors, such as anthropogenic disturbance, can also influence the formation and change in the spatial patterns of species [31]. Here, we examined a deciduous broad-leaved forest, primarily consisting of mixed oak forests, located in a minimally human-disturbed area at the mountain’s base. Consequently, our focus was on assessing the impact of biological factors on the forest’s distribution pattern.

Environmental heterogeneity is considered a significant factor influencing the spatial distribution patterns of vegetation, as suggested in earlier studies [32,33]. In our investigation of the mixed forests of Yushan, we specifically examined its impact on the distribution of four oak species. Our findings indicate that environmental heterogeneity substantially affects certain species, particularly the *Q. variabilis* population. This species showed a discrete distribution pattern when environmental factors were accounted for, differentiating it from others like *Q. acutissima*, which exhibited an aggregated distribution in the 0–14 m range. However, the remaining two populations of the *Quercus* group (*Q. fabri* and *Q. serrata* var. *Brevipetiolata*) showed relatively little impact. Drawing upon previous studies [34,35,36], we hypothesize that the species in the *Cerris* group, especially *Q. variabilis*, are more susceptible to environmental heterogeneity than those in the *Quercus* group, likely due to the latter’s broader habitat range.

Delving further into the distribution patterns through the lens of the IPP model, we noted that even after controlling for environmental heterogeneity, the populations of the four oak species still exhibited an aggregated distribution. This pattern is potentially attributable to hybridization processes [29]. It appears that the *Quercus* group, in comparison to the *Cerris* group, has a wider aggregated distribution, suggesting a higher acorn dispersal capability. This could result in asymmetrical introgression directions and contribute to spatial and temporal variances in the distribution patterns of these species [29].

After a comprehensive evaluation of the CSR and IPP models in the context of our data, we have chosen the IPP model as our primary analytical tool. This model’s capacity to integrate environmental heterogeneity into its analysis makes it particularly suited for our study. In examining the oak populations’ distribution with the IPP model, we observed a tendency towards random distribution patterns beyond a 16-m range, as opposed to the strong aggregation indicated by the CSR model. This distinction is most notable in species like *Q. acutissima* and *Q. variabilis*, where the IPP model’s ability to capture the influence of various environmental factors on distribution patterns is clearly demonstrated.

We can broadly classify interspecific distribution patterns into three types: discrete distributions resulting from interspecific competition, random distributions devoid of any interspecific ties, and aggregated distributions resulting from interspecific interactions. In nature, species have a less random distribution, with the majority having distinct and aggregated distributions. We investigated six interspecific relationships: *Q. acutissima*–*Q. variabilis*, *Q. acutissima*–*Q. fabri*, *Q. acutissima*–*Q. serrata var. brevipetiolata*, *Q. variabilis*–*Q. fabri*, *Q. variabilis*–*Q. serrata var. brevipetiolata*, and *Q. fabri*–*Q. serrata var. brevipetiolata*. The six pairs of interspecific relationships included the distribution pattern between the same group and different groups. Apart from *Q. acutissima*–*Q. variabilis*, the *K*_12_(r) statistical function identified a clustered distribution of interspecific relationships in the five species pairs. These outcomes showed the tight relationships and co-existence of the four oak species in this deciduous broad-leaved forest. *Q. acutissima* and *Q. variabilis* dispersion patterns predicted more intra-group rivalry than inter-group competition. It is conceivable that this has much to do with their reproductive isolation.

For example, there is a gradual trend toward dispersion and a decrease in aggregation over long distances between populations of the closely related species *Q. acutissima* and *Q. variabilis*, as well as between *Q. serrata* var. *brevipetiolata* and *Q. fabri* (Figure 2a,f), with closely related species lacking reproductive isolation and occupying different ecological niches through functional differences to reduce introgression. There is reproductive isolation between distantly related species, and interspecific gene flow is low, hence the aggregated state. *Q. acutissima* and *Q. serrata* var. *brevipetiolata* populations were less aggregated as distance increased, as seen in Figure 2c. We considered the existence of greater gene exchange between these two species than between other distantly related species, gradually dispersing to maintain their populations’ coexistence [37].

### 3.2. Influence of Leaf Functional Traits on Coexistence in Oak Species

In most studies, we have found that ecological mechanisms promote the long-term coexistence of multiple species of oak. For example, the influence of leaf traits on microbial communities or soil nutrient dynamics facilitates the establishment of new colonies and reduces competition by delineating ecological niches at fine spatial scales [38]. *Q. deserticola* and *Q. castanea* have coexisted in Mexico for a long time, with the former’s leaves being long-lived but with a low rate of nutrient uptake, and the latter’s leaves being short-lived but with a high rate of nutrient uptake [39]. Soil fertility is higher in mixed stands of these two oak populations than in single stands, thus increasing their survival and development chances. In this study, we found significant differences in TC, TP, and TK among the four oak species, suggesting that species are likely to influence the microbial community or soil nutrients through their nutrient uptake, thus delineating their respective ecological niches. The three-leaf functional attributes (TC, TP, and TK) correspond with spatial location aggregation, whereas TN correlates with spatial location dispersion, showing that the four oak species have varied nutritional sensitivity.

Individuals were clustered based on their DBH into two classes: large and small DBH. This aggregated distribution is more conducive to the group effect, increasing interspecific competitiveness and resistance to invasive alien species. Consequently, oak has become the dominating tree in the area, which may be attributed to limited seed dispersal or other factors [40]. In addition, the differences in leaf functional traits allowed them to have their own ecological niches in fine-scale spaces, which helps to sustain population and biodiversity levels at normal levels. *Q. fabri* and *Q. variabilis* individuals with high leaf TC clustered together, whereas *Q. fabri*, *Q. variabilis*, and *Q. acutissima* individuals with low leaf TP concentrations clustered together, indicating that *Q. fabri* and *Q. variabilis* have similar ecological niches based on leaf traits TC and TP, whereas *Q. acutissima* and *Q. serrata var. brevipetiolata* existed in a different ecological niche. TN local distribution is more fully reflective of the partitioning of resources by oak trees at a fine scale. Individuals with high leaf TN concentrations are surrounded by individuals with low leaf TN, and vice versa. However, the spatial distribution of TK content was not very obvious.

In the context of broader ecological theories, our findings offer intriguing insights into the spatial distribution and nutrient element variations in the four oak species in Yushan’s mixed forest. The observed patterns of spatial distribution, particularly the interspecific relationships and DBH distributions, align with the theory of niche differentiation. This principle posits that coexisting species tend to occupy distinct ecological niches, thus reducing direct competition and facilitating biodiversity. Our study reveals that each oak species exhibits unique spatial and nutritional characteristics, suggesting a form of niche partitioning that supports their coexistence. Additionally, the significant nutrient element variations observed among the species resonate with the concept of resource partitioning, where different species utilize varying nutritional strategies to coexist in a shared environment. Our study indicated the interplay of competition and cooperation among oak species. However, for a more comprehensive understanding of the competition and cooperation among oak populations, further research and analysis is needed, including consideration of other factors such as soil characteristics, light conditions, water use efficiency, and long-term monitoring of population dynamics and growth status. Such findings challenge and extend the classical models of forest ecology, indicating a need to consider the nuanced interactions and adaptations of species within mixed forests.

Although our research reveals the complex dynamics of competition and cooperation among oak species, it is important to note that our sampling approach may not have been sufficiently comprehensive to include all other tree species in the study area. Interspecific interactions are a crucial factor influencing species distribution models. Therefore, in future research, we plan to consider this factor to gain a more comprehensive view of the ecosystem’s structure and function. This will help us deepen our understanding of the ecological roles and relationships of oak species in a broader forest context, thus more comprehensively assessing their contribution to and impact on the forest.

## 4. Materials and Methods

### 4.1. Study Area

Yushan, located in the northwest of Changshu City (31°36′ N, 120°40′ E, 263 m), Jiangsu Province, is a semi-natural forest characterized by its unique geographical and climatic conditions. It is the longest and highest mountain extending on the forefront plain of the Yangtze River Delta. The ridge extends northwestward for 6,400 meters. The highest peak, Wanghaidun, has an elevation of 261 meters. The mountain stands tall on the vast plain. Positioned at the edge of the northern subtropical monsoon climate zone, it experiences an average annual temperature of 15.6 °C and receives an average annual rainfall of 1062.5 mm, with a significant concentration of rainfall during summer. Yushan’s geological lithology is primarily characterized by quartz sandstone and mudstone from the Maoshan Group and the Wutong Formation, overlaid with acidic weathering materials and colluvial deposits. These rocks and sediments, through prolonged weathering processes, have formed yellow-brown soils, presenting an acidic environment with a pH value between 5 and 6. The forest is diverse, comprising mainly mixed evergreen deciduous broad-leaved forests, deciduous broad-leaved forests, coniferous forests, and scrub. Notably, the deciduous broad-leaved forests are dominated by four oak species including *Quercus acutissima*, *Q. variabilis*, *Q. fabri*, and *Q. serrata* var. *brevipetiolata*, underscoring the ecological importance of oaks in this region.

### 4.2. Plant Materials and Measurements

**Figure 6:**
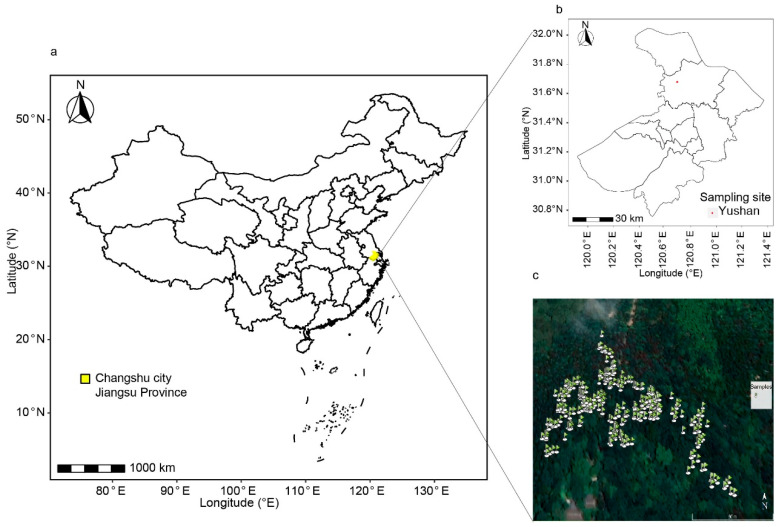
The geographical locations of the sampling site and distribution of sampled trees. (**a**) The sampling site in Jiangsu Province, China; (**b**) The sampling site in Yushan, Changsu City; (**c**) GPS information of samples.

In July 2018, we sampled 300 oak individuals in a mixed oak woodland, measured their height (H) and DBH, and recorded their geographic coordinates using GPS (V990) (Figure 6 and Table 4). From each individual within the 80 m × 250 m study area (Figure 6), three replications of five mature and healthy leaves were collected from different branches in different directions, quickly preserved in silica gel, and dried at 60 °C. Samples were identified by leaf morphology and preserved in Nanjing Forestry University. We crushed and passed samples through a 100-mesh sieve for ecological stoichiometry measurements. From each sample, 0.2 g was digested with H_2_SO_4_ and H_2_O_2_. and total nitrogen (TN), total phosphorus (TP), and total potassium (TK) concentration were determined by the Kjeldahl method, the colorimetric method (ultraviolet spectrophotometer), and the flame photometry method, respectively. Total carbon (TC) concentration was determined using the potassium dichromate volumetric method, using 0.02 g per sample [41].

### 4.3. Data Analysis

#### 4.3.1. Point Pattern Analysis

GPS information of the 300 sampled oak trees was first converted to Cartesian coordinates using the R packages ‘SoDA’ (version 4.1.0). Subsequently, Point Pattern Analysis (PPA) was conducted, employing the R-Spatstat package. We used the univariate Ripley’s *K*(*r*) and bivariate Ripley’s *K*_12_(*r*) functions for the analysis of single oak populations and interspecific relationship patterns, respectively. Ripley’s *K*-function measures the spatial distribution pattern of points within a study area. It quantifies the ratio of the number of desired points within a circle centered at any point in the study area, with a radius of ‘*r*’, to the density of points within the entire study area. In essence, it assesses whether points are randomly dispersed, clustered, or regularly spaced across the study area [42]:

(1) $$K_{\left(t\right)}=\lambda^{-1}$$

where *t* represents any value greater than 0 and *λ* is the density (number per unit area) of events. In practice, we estimated the pattern of individual population distributions using Diggle’s (1983) formula [43]:

(2) $$K_{\left(r\right)}=n^{-2}A\sum_{i=1}^{n}\sum_{j=1}^{n}\frac{1}{Wij}I_{r}\left(d_{i,j}\right)$$

where *A* is the sample area, and *n* is the total number of points (number of individuals of the species). *d_ij_* is the distance between two points *i* and *j*, and *I_r_* is the indicator function. The weight function, *W*_ij_, provides the edge correction.

The following equation was used to analyze the relationship between any two oak populations (bivariate PPAs) [43]:

(3) $${K_{12}\left(r\right)=n}_{1}n_{2}^{-2}A\sum_{i=1}^{n1}\sum_{j=1}^{n2}\frac{1}{Wij}I_{r}\left(d_{ij}\right)\;\;\;$$

where *n*_1_ and *n*_2_ are the numbers of individuals of species one and species two, respectively, *i* and *j* represent the number of individuals of species one and species two, respectively, and the rest are as in (2). When *K*_obser_ (*r*) > *K*_theo_ (*r*), the species distribution shows aggregation; when *K*_obser_ (*r*) = *K*_theo_ (*r*), the species shows a random distribution; and when *K*_obser_ (*r*) < *K*_theo_ (*r*), the species shows a dispersed distribution.

For PPA, different null models were chosen for the two functions. The univariate *K*(*r*) function is chosen to compare both the Complete Spatial Randomness (CSR) model and the Inhomogeneous Poisson Process (IPP) model. The bivariate *K*_12_(*r*) function was only chosen for the Complete Spatial Randomness (CSR) model. The upper and lower packet traces were calculated using 199 Monte-Carlo fit tests, i.e., 99% confidence intervals [44].

#### 4.3.2. Breast Diameter and Nutrient Element Analysis

The classification of all species was meticulously conducted following Zhongxiang Qu’s method, which delineates standing trees and seedlings into four distinct size classes based on diameter at breast height (DBH): ≤2.5 cm; >2.5 cm and ≤7.4 cm; >7.4 cm and ≤22.5 cm; and >22.5 cm [45]. Subsequently, the breast diameter measurements were systematically categorized for quantitative analysis. To ensure the integrity of the dataset, normality tests were administered on the concentrations of total carbon (TC), total nitrogen (TN), total phosphorus (TP), and total potassium (TK). In order to discern the variations in TC, TN, TP, and TK across different species, a one-way analysis of variance (ANOVA) was conducted, with further *post hoc* tests being employed for detailed pairwise comparisons of these nutrient elements and their ratios among the species. Moreover, to capture the underlying relationships between these variables, a Principal Component Analysis (PCA) was performed, utilizing the correlation coefficients of TC, TN, TP, and TK. This methodological approach ensures a coherent and logical progression in analyzing and interpreting the data concerning nutrient elements within various species.

#### 4.3.3. Spatial Autocorrelation Analysis

Utilizing spatial datasets, an adjacency matrix is constructed through the application of functions contained within the ‘spdep’ package. This adjacency matrix is then utilized to derive spatial weights that quantify the interactions between each spatial unit and its adjacent counterparts. Subsequently, the weights associated with each solid surface are normalized utilizing a standardized model. The statistical variance is computed, and the significance of the global Moran’s I statistic is evaluated through the application of a random hypothesis testing approach, as delineated in reference [46]:

(4) $$I=\frac{n\;\sum_{i=1}^{n}\sum_{j=1}^{n}w_{i,j}z_{i}z_{j}}{S_{0}\cdot \sum_{i=1}^{n}z_{i}^{2}}$$

where $z_{i}$ is the deviation of an attribute for feature i from its mean, $w_{i,j}$ is the spatial weight between features *i* and *j*, *n* is equal to the total number of features, and *S*_0_ is the aggregate of all the spatial weights. Moran’s *I* value is distributed between [–1, 1]. Moran’s I > 0 indicates a positive spatial correlation: the larger the value, the more obvious the spatial correlation; Moran’s I < 0 indicates a negative spatial correlation: the smaller the value, the greater the spatial variation; Moran’s I = 0, indicates no correlation.

To better understand the distribution pattern of the four oak species in mixed forests, DBH distribution and nutrient elements of all oak trees were examined locally at the same time, and scatter plots were drawn. The *x*-axis is used as the variable (DBH, TC, TN, TP, and TK) and the *y*-axis is set to the spatially weighted sum of the nearest neighbor values (spatially lagged values). Global Moran’s I is expressed as a linear relationship between *x* and *y* in one diagonal line as follows [47]:

(5) $$I_{i}=\frac{\left(y_{i}-\bar{y}\right)\sum_{j=1}^{n}\omega_{i,j}\left(y_{i}-\bar{y}\right)}{\frac{\sum_{i=1}^{n}{\left(y_{i}-\bar{y}\right)}^{2}}{n}}$$

All analyses were conducted in R [48].

## 5. Conclusions

In our study of the mixed oak forest in Yushan China, we have highlighted the significance of leaf functional traits, particularly nutritional elements. These elements, crucial for plant growth and development, also serve as vital functional traits that significantly contribute to understanding plant coexistence. Our findings reveal notable variations in C, N, P, and K levels among the four oak species (*Quercus acutissima*, *Q. variabilis*, *Q. fabri*, and *Q. serrata* var. *brevipetiolata*), indicating distinct ecological strategies driven by both genetic and environmental factors. This mixed forest, characterized by a state of hybridization, shows that the variability in these nutrient elements is a result of the interplay between genetics and the environment. Future research should focus on analyzing the genetic links to these nutritional elements, enhancing our understanding of how ecological dynamics and biological evolution combine to maintain species coexistence in mixed forest ecosystems. This approach is crucial for developing effective forest management and conservation strategies.

## Figures and Tables

**Table 1:** The summary of DBH in four oak populations.

Species	Max (cm)	Min (cm)	Average (cm)
*Q. acutissima*	18.50	1.00	6.50
*Q. variabilis*	36.60	0.50	15.79
*Q. fabri*	38.40	1.00	12.37
*Q. serrata* var. *brevipetiolata*	36.70	0.50	14.89
All species	38.40	0.50	13.00

**Table 2:** The summary of TC, TN, TP, and TK concentrations of leaves in four oaks.

Species	TC (mg g^−1^)		TN (mg g^−1^)		TP (mg g^−1^)		TK (mg g^−1^)	
	Mean ± SD	Fold	Mean ± SD	Fold	Mean ± SD	Fold	Mean ± SD	Fold
*Q. acutissima*	313.41 ± 51.21 ^a^	2.26	17.89 ± 6.11 ^a^	4.00	0.73 ± 0.25 ^b^	6.08	4.47 ± 2.35 ^c^	11.89
*Q. variabilis*	312.66 ± 73.55 ^a^	4.38	17.69 ± 5.26 ^a^	11.05	0.87 ± 0.32 ^a^	6.13	5.34 ± 2.62 ^ab^	12.63
*Q. fabri*	290.56 ± 74.45 ^ab^	4.89	18.64 ± 5.78 ^a^	5.24	0.88 ± 0.33 ^a^	10.15	5.57 ± 2.43 ^a^	8.43
*Q. serrata* var. *brevipetiolata*	272.52 ± 75.22 ^b^	3.64	18.14 ± 5.69 ^a^	6.52	0.80 ± 0.44 ^ab^	13.04	4.60 ± 1.99 ^bc^	9.68
All species	296.85 ± 72.59	5.01	18.12 ± 6.78	11.58	0.83 ± 0.34	15.00	5.10 ± 2.42	15.68

Note: Significant differences in nutrient elements between species were determined using the multiple comparison (LSD) method. These differences among groups are indicated by the letters a, b and c. “Fold” refers to the ratio of the maximum value to the minimum value.

**Table 3:** Spatial intercorrelation between five indicators.

	Moran’s *I*	Expectation	Variance	Standard Deviate	*p*-Value
DBH	0.5583	−0.0033	0.0005	24.1678	<0.000 ***
TC	0.2253	−0.0033	0.0005	9.8785	<0.000 ***
TN	−0.0121	−0.0033	0.0005	−0.3952	0.6536
TP	0.0963	−0.0033	0.0005	4.3324	<0.000 ***
TK	0.0253	−0.0033	0.0005	1.2352	0.1084

*p* < 0.001 ***.

**Table 4:** Information of samples in oaks mixed forest.

Population	Code	Section	Altitude (m)	Site	Latitude (N)/ Longitude (E)	Sample Size
*Q. acutissima*	ML	*Cerris*	263	Yushan	N31°36′/E120°40′	48
*Q. variabilis*	SP	*Cerris*				89
*Q. fabri*	BL	*Quercus*				96
*Q. serrata* var. *brevipetiolata*	DB	*Quercus*				67
Total						300

## Data Availability

Data are contained within the article.
